# IL-17A contributes to perioperative neurocognitive disorders through blood-brain barrier disruption in aged mice

**DOI:** 10.1186/s12974-018-1374-3

**Published:** 2018-11-30

**Authors:** Pengfei Ni, Hongquan Dong, Yiwei Wang, Qin Zhou, Mengmeng Xu, Yanning Qian, Jie Sun

**Affiliations:** Department of Anesthesiology, The First Affiliated Hospital of Nanjing Medical University, Jiangsu, 210029 People’s Republic of China

**Keywords:** IL-17A, Blood-brain barrier, Neuroinflammation, Neurodegeneration, Perioperative neurocognitive disorders

## Abstract

**Background:**

Perioperative neurocognitive disorders (PND) occur frequently after surgery, especially in aged patients. Surgery-induced neuroinflammation and blood-brain barrier (BBB) dysfunction play a crucial role in the pathogenesis of PND. Interleukin-17A (IL-17A) increases after surgical stress and will be involved in BBB dysfunction. However, the effect of IL-17A on BBB function during PND remains poorly understood.

**Methods:**

Male wild-type C57BL/6J mice (15 months old) received tibial fracture surgery and fixation to establish the PND model. All the mice were injected intraperitoneally with an IL-17A-neutralizing antibody (Abs) or isotype-control Abs 30 min before tibial fracture surgery. Animal behaviour tests conducted 24 h after surgery included the contextual fear conditioning and Y maze tests. Serum and hippocampus IL-17A levels and hippocampus IL-6 and IL-1β levels were detected by ELISA. BBB function was detected by Evans blue (EB) test. Hippocampus matrix metalloproteinase-2 (MMP-2)- and MMP-9-positive cells were detected by immunohistochemistry. Hippocampus albumin, occludin, claudin-5 and IL-17A receptors were detected by Western blot. For the in vitro experiment, bEnd.3 cells were incubated with IL-17A. Cell IL-17A receptors were detected by immunofluorescence. Cellular MMP-2, MMP-9, occludin, and claudin-5 were detected by Western blot.

**Results:**

Tibial fracture surgery promoted memory impairment, increased levels of IL-17A and IL-17A receptors, inflammatory factor production and BBB dysfunction. IL-17A Abs inhibited this effect, including improving memory function, decreasing inflammatory factor production and alleviating BBB disruption, indicated by decreased tight junctions (TJs) and increased MMPs after surgery. The in vitro study suggested that recombinant IL-17A could upregulate the expression of IL-17A receptors, decrease TJs and increase the level of MMPs in bEnd.3 cells.

**Conclusions:**

Our results suggested that IL-17A-promoted BBB disruption might play an important role in the pathogenesis of PND.

**Electronic supplementary material:**

The online version of this article (10.1186/s12974-018-1374-3) contains supplementary material, which is available to authorized users.

## Background

Perioperative neurocognitive disorder (PND) is a recently recommended overarching term for cognitive impairment in the preoperative or postoperative period and is associated with increased mortality [1]. It is characterized as a decline in cognitive functions including memory, attention, information processing and cognitive flexibility [2]. It has been reported that advanced age is one of the major risk factors [3]. However, the pathogenesis underlying PND still remains unknown. Increasing evidence suggests that neuroinflammation is a key contributor to PND [4, 5]. Li et al. have shown that early postoperative inflammation is important for neurodevelopment-associated outcomes 2 years after the Norwood procedure [6]. Surgical trauma leads to an increase in systemic inflammatory mediators, such as Il-β and IL-6, which have been shown to influence neuroinflammatory cascade in the brain and impair cognitive function [7]. In addition, the ageing process itself has been associated with enhanced neuroinflammatory response and more pro-inflammatory cytokines [8, 9].

The BBB is an important structure that separates the central nervous system (CNS) from the periphery, protects the brain from harm and maintains homeostasis in the brain. BBB disruption allows the entry of neurotoxic debris, cells and pathogens and contributes to inflammatory and immune responses in the CNS [10]. BBB breakdown has been found in several autoimmune and neurodegeneration diseases, such as Alzheimer’s disease (AD), Parkinson’s disease (PD) and multiple sclerosis (MS) [11–13]. Some research has shown age-dependent BBB breakdown in the hippocampus, which may be associated with cognitive decline during ageing [14, 15]. Recent findings have suggested that BBB disruption is prevalent in the first 24 h after surgery, and dysfunction of the BBB may facilitate the passage of peripheral immune cells and mediators to the brain, which promotes brain inflammation and neuronal damage [16, 17].

IL-17 is mainly secreted by T helper 17 (Th17) cells, and the IL-17 family includes six members designated IL-17A-F, among which IL-17A is the most important member. IL-17A is emerging as a crucial pro-inflammatory cytokine contributing to the occurrence and development of several CNS inflammatory diseases [18, 19]. Some researches revealed that some clinical drugs to alleviate neuroinflammation in multiple sclerosis worked through regulating Th17 cell differentiation [20, 21]. Our previous data indicated that IL-17A was involved in LPS-induced cognitive impairment in aged rats [22]. What is more, IL-17 levels in the supernatant of drainage fluid from patients received colectomies via laparotomy rapidly reached the maximum 1–2 days after surgery [23]. In an experimental autoimmune encephalomyelitis (EAE) model, IL-17A induced BBB breakdown by reactive oxygen species (ROS) production and a decrease in tight junctions (TJs) [24]. In consideration of the role of IL-17A in CNS inflammation and the impact on BBB permeability in the EAE model and to further decipher the role of IL-17A on BBB function in PND, we used a tibial fracture surgical model in aged mice. Human anti-IL-17A antibody has been tested clinically and shown proven efficacy and safety in chronic inflammatory diseases [25–27]. What is more, an in vitro experiment showed that secukinumab, the FDA-approved anti-IL-17 antibody, could rescue the neuronal death from Parkinson’s disease patients [28]. In view of the clinical study of human anti-IL-17 antibody, it is of great clinical significance and application value to explore the role of IL-17A in PND. We hypothesized that surgical trauma induces the release of IL-17A and drives the BBB breakdown, promoting the inflammatory response in the CNS and finally leading to cognitive dysfunction.

## Materials and methods

### Reagents

Dulbecco’s modified Eagle’s medium (DMEM), 0.25% trypsin-EDTA solution and foetal bovine serum (FBS) were purchased from Gibco-BRL (Grand Island, NY, USA). Mouse IL-17A and isotype-control Abs were purchased from R&D Systems (Minneapolis, MN, USA). Monoclonal mouse anti-MMP-2, monoclonal mouse anti-MMP-9 and monoclonal rabbit anti-occludin were purchased from Abcam (Hong Kong, China). Monoclonal mouse anti-claudin-5 antibody was purchased from Invitrogen (Invitrogen, USA). Recombinant IL-17A protein and Fluoroshield mounting medium with 4,6-diamidino-2-phenylindole (DAPI) were purchased from Abcam (Hong Kong, China). The mouse IL-17A ELISA kit, IL-6 ELISA kit and IL-1β ELISA kit were obtained from R&D Systems (Minneapolis, MN, USA).

### In vivo studies

#### Animals

Male wild-type C57BL/6J mice (15 months old) were purchased from Jinling Hospital of Nanjing University. All mice were housed in groups of five animals per cage with free access to food and water under standard laboratory conditions, a 12/12-day/night cycle and a constant room temperature of 22 ± 1 °C. All experimental procedures were approved by the Nanjing Medical University Animal Care and Use Committee and performed according to the Guide for the Care and Use of Laboratory Animals of the National Institutes of Health of the United States.

#### Design and treatment groups

**Fig. 1:**
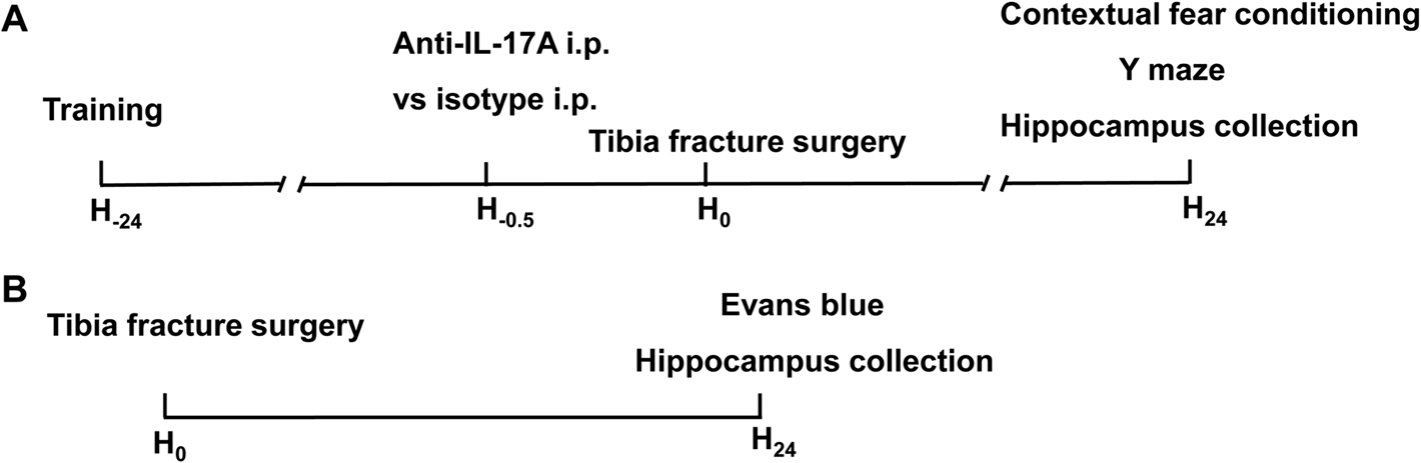
Study design. **a** Experiment 1: TFC test training was performed 1 day before surgery. Mice in the Con+anti-IL-17A group received i.p. anti-IL-17A Abs, and the Con+isotype group received an equivalent dose of isotype-control Abs 30 min before surgery. Behavioural tests included TFC and the Y maze performed 24 h after surgery, and then the hippocampi were collected. **b** Experiment 2: After behavioural tests, the mice received an intravenous injection of EB, and the hippocampi were collected for EB extravasation detection

The mice were randomly allocated to 4 groups with 12 mice in each group: (i) control group (Con group), (ii) tibial fracture surgery group (Sur group), (iii) tibial fracture surgery following i.p. injection of anti-IL-17A Abs group (Sur+anti-IL-17A group) and (iv) tibial fracture surgery following i.p. injection of isotype-control Abs group (Sur+isotype group). Mice in the Sur+anti-IL-17A group received 3 mg/kg anti-IL-17A Abs, and those in the Sur+isotype group received 3 mg/kg isotype-control Abs 30 min before surgery. One day before surgery, mice were performed behavioural training, and behavioural test was performed 1 day after surgery. Following behavioural test, Evans blue (EB) extravasations were detected and hippocampus tissues and serums were collected. The study design is briefly illustrated in Fig. 1.

#### Surgery

Following isoflurane anaesthesia (2.0% inspired concentration in 40% FiO2), open tibial fracture surgery was performed as previously described [29, 30]. Briefly, the left tibia was shaved and disinfected, and a middle incision lateral to the tibia was made, followed by insertion of an intramedullary fixation pin in the bone marrow cavity. Then, we stripped the periosteum and performed an osteotomy at the junction of the middle and upper third of the tibia under direct vision. Finally, the wound was sterilized and sutured, and analgesia (buprenorphine 0.1 mg/kg subcutaneously) was injected subcutaneously. During the procedure, a sterile surgical field was maintained and a heating pad was used to maintain the body temperature.

### Behavioural analyses

#### Contextual fear conditioning

Contextual fear conditioning has been used to assess learning and memory in rodents. Mice were trained to associate the environment (context) with the conditional stimulus (tone) and the unconditional stimulus (foot shock). Mice were placed in the conditioning chamber and allowed exploration of the environment for 100 s. Then, the conditional stimulus, an auditory cue (65 dB, 3 kHz), was presented for 20 s, and the unconditional stimulus, a foot shock (0.7 mA, 0.5 s), was performed after termination of the tone. After an interval of 100 s, the procedure was repeated. Mice were removed from the chamber 30 s after the procedure. Finally, we performed a contextual assessment 24 h after surgery but with no cues (tone or shock) in the same chamber. Freezing behaviour (the absence of all movement except for respiration) was recorded for 300 s by video and analysed by software (Xeye Fcs, Beijing Macro Ambition S&T Development Co., Ltd., Beijing, China). All tests were performed blindly by an investigator.

#### Y maze test

The Y maze was composed of three arms (regions I–III, 30 cm l × 5 cm w × 20 cm h) with a lamp at the distal end of each arm, which converged to the equilateral triangular central area. The safe region was illuminated, and the other regions had electrical foot stimulation (25 V). First, each mouse was placed randomly at the end of one arm and allowed to explore the environment for 3 min. Then, the test was started, and the illuminated arm (safe region) served as the new starting area. Next, we changed the orientation of the safe region and stimulation region randomly. The standard of success (learned) was reaching the safe region within 10 s. After each stimulation, we waited for the mouse to reach the illuminated arm before the next stimulation and recorded which arm was chosen to be the new starting area. If nine responses were correct in ten consecutive stimulations (9/10 standard), the mice were considered as having reached learning criterion. We recorded the total number of stimulations to reach criterion as the learning ability during training. All tests were performed blindly by an investigator.

#### EB extravasation

BBB permeability was evaluated by EB (EB; Sigma) extravasation. First, 2% EB (4 ml/kg) was injected intravenously and allowed to circulate for 1 h. Following anaesthesia, mice were perfused with 20 ml normal saline through the left ventricle. The hippocampus was extracted and immersed in formamide (Sigma-Aldrich) for 72 h at 37 °C. Then, formamide was centrifuged at 12,000*g* for 20 min. The absorbance of the hippocampus was measured at 632 nm (BioTek, Vermont, USA). The EB content was calculated from the standard EB curve to measure BBB permeability.

#### Immunohistochemistry

Mice were anaesthetized with 1% pentobarbital (10 μl/g) and perfused with 0.9% saline and 4% cold paraformaldehyde successively. The brains were harvested and fixed with 4% paraformaldehyde at 4 °C overnight. The brain sections were prepared using a cryostat. Immunohistochemistry was processed as described next. Following incubation for 1 h in 10% bovine serum albumin with 0.3% Triton X-100 in 0.01 M PBS, the sections (30 μm) were incubated with anti-MMP-2 monoclonal antibody (1300) and anti-MMP-9 monoclonal antibody (1:500) overnight at 4 °C, and the sections were incubated with secondary antibody for 2 h. Immunostaining was visualized using 3,3′-diaminobenzidine. The sections were then counterstained with haematoxylin. Positive cells were visualized by adding DAB to the sections. Images of the immunostaining were digitally captured by using a Leica 2500 microscope.

##### ELISA

The level of IL-17A in serum and hippocampal tissue extracts and IL-6 and IL-1β levels in hippocampal tissue extracts were measured with ELISA kits from R&D Systems. Data were collected and analysed using the Luminex-200 System version 2.3.

### In vitro studies

#### Cell culture

Immortalized BALB/C BECs [bEnd.3; American Type Culture Collection (ATCC), Manassas, VA, USA] derived from brain endothelial cells of a primary mouse were used as the BBB model. Cells were cultured in DMEM supplemented with 10 % foetal bovine serum and penicillin-streptomycin (0.6 × 105 μl^−1^). Cells were maintained at 37 °C and 5 % CO_2_ air atmosphere, and the medium was changed every 48 h.

#### Recombinant IL-17A treatment

The treatment was performed when cells were in a logarithmic phase of growth. bEnd.3 cells were seeded (1 × 10^6^ cells) in 5 cm × 5 cm flasks and incubated for 24 h at 37 °C and in 5% CO_2_ humidified atmosphere. The cells were treated with recombinant IL-17A (10, 50 and 100 ng/ml) for 24 h and collected.

#### Immunofluorescence

To evaluate the expression of IL-17A receptors in bEnd.3 cells, cells were fixed with 4% paraformaldehyde for 30 min. Unspecific binding was blocked by 5% BSA and 0.1% Triton X-100 solution at room temperature for 1 h. Cells were incubated with Rabbit anti-IL-17A receptor polyclonal antibody (1:50) in the blocking solution at 4 °C overnight. After three washes with PBS, the cells were incubated with corresponding FITC-conjugated goat anti-rabbit IgG (1:200) at 37 °C for 1 h, and the nuclei were stained with DAPI. Fluorescent images were acquired using a confocal microscope.

#### Western blot

Hippocampal tissues and bEnd.3 cells were homogenized in RIPA lysis buffer containing 20 mM Tris, 150 mM NaCl, 1 mM EDTA, 1% Triton X-100, 1.5 μg/ml leupeptin, and 1 mM phenylmethylsulfonyl fluoride (PMSF). The samples were centrifuged at 12,000×*g* (4 °C) for 20 min, and the supernatants were harvested. Protein concentration was determined using a BCA kit. Proteins were separated by 10% sodium dodecyl sulfate-polyacrylamide gel electrophoresis (SDS-PAGE) and transferred to a PVDF microporous membrane (Millipore, USA). The membranes were incubated with 5% non-fat milk at room temperature for 1 h and incubated overnight at 4 °C with primary antibodies: polyclonal rabbit anti-IL-17A receptor (1:500), monoclonal mouse anti-MMP-2 (1:1000), monoclonal mouse anti-MMP-9 (1:500), monoclonal mouse anti-claudin-5 (1:500) and monoclonal rabbit anti-occludin (1:1000). An antibody against GAPDH (1:1000) was also used as an internal standard. The membranes were incubated with goat anti-mouse secondary or goat anti-rabbit secondary antibodies at room temperature for 1 h and then detected with a chemiluminescent substrate. The relative densities of the protein bands were visualized and analysed by Image Lab software (Bio-Rad, Richmond, CA, USA) and NIH ImageJ software (Bethesda, MD, USA), respectively.

### Statistical analysis

All values are presented as the mean ± SEM. The significance of the differences between groups was tested using a one-way ANOVA followed by post hoc least significant difference tests. *P* < 0.05 was considered statistically significant.

## Results

### Tibial fracture surgery-induced cognitive impairment and treatment with anti-IL-17A Abs alleviated this effect

**Fig. 2:**
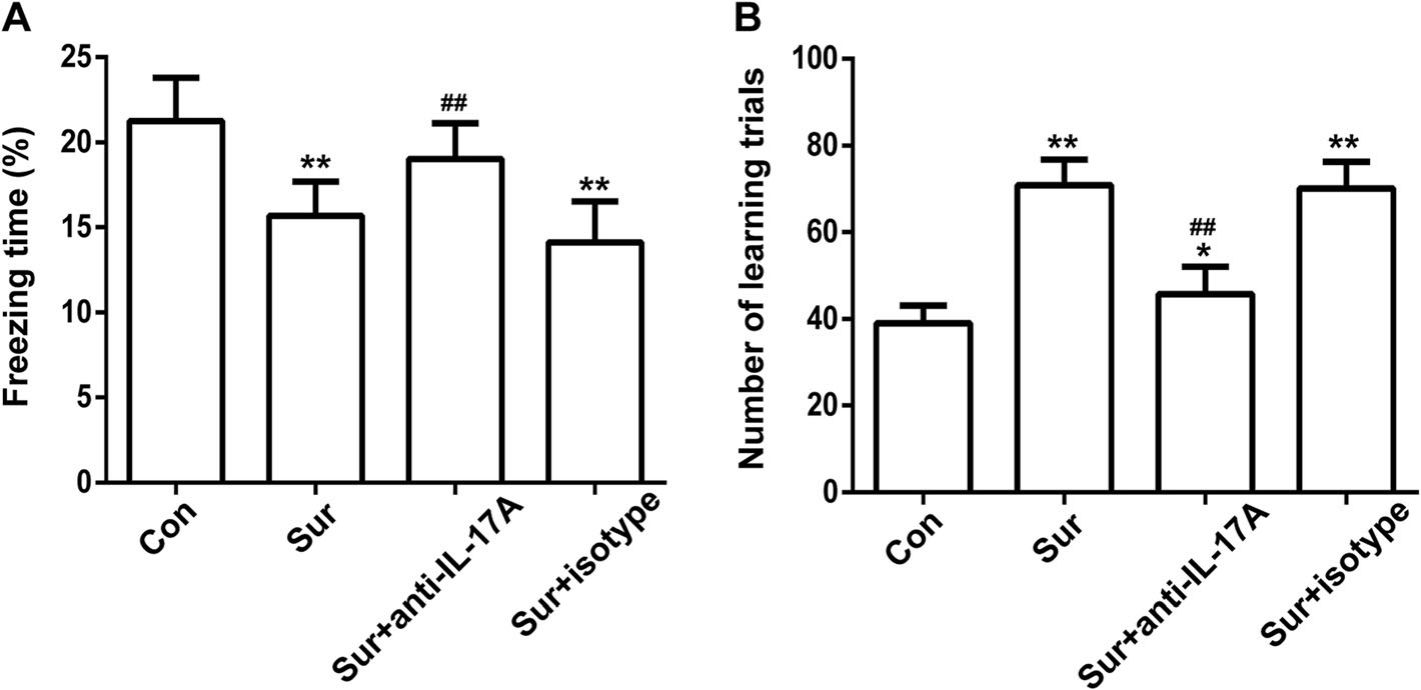
Tibial fracture surgery-induced cognitive impairment and treatment with anti-IL-17A Abs alleviated this effect. The freezing time in the TFC test (**a**) and the number of learning trials in the Y maze test (**b**) were recorded to analyse the cognitive changes. *P < 0.05 and **P < 0.01 versus the Con group. ^##^*P* < 0.01 versus the Sur group. Data are presented as the mean ± SEM (*n* = 12)

To investigate the effect of surgery on cognitive function and whether IL-17A was involved, mice were injected i.p. with anti-IL-17A Abs or isotype-control Abs 30 min prior to tibial fracture surgery. Then, 24 h after surgery, we assessed learning and memory with behavioural tests. As shown in Fig. 2, compared with controls, the mice in the surgery group had a significant decrease in freezing time and increased number of learning trials (freezing: Sur group 15.67 ± 0.59 versus Con group 21.26 ± 0.73, *P* < 0.001; number of learning trials: Sur group 64.83 ± 1.93 versus Con group 39.00 ± 1.39, *P* < 0.01), which indicates a reduction in cognitive function. Treatment with anti-IL-17A Abs increased freezing time and decreased the number of learning trials compared to surgery (freezing: Sur+anti-IL-17A group 19.04 ± 0.61 versus Sur group 15.67 ± 0.59, *P* < 0.001; number of learning trials: Sur+anti-IL-17A group 45.83 ± 1.82 versus Sur group 64.83 ± 1.93, *P* < 0.01). Treatment with isotype-control Abs had no effect on learning and memory. Detailed training and learning data of contextual fear conditioning test was included in Additional file 1. These results suggested that tibial fracture surgery-induced cognitive impairment could be alleviated by treatment with anti-IL-17A Abs.

### Tibial fracture surgery increased serum and hippocampus IL-17A levels and hippocampus IL-17A receptors expression

**Fig. 3:**
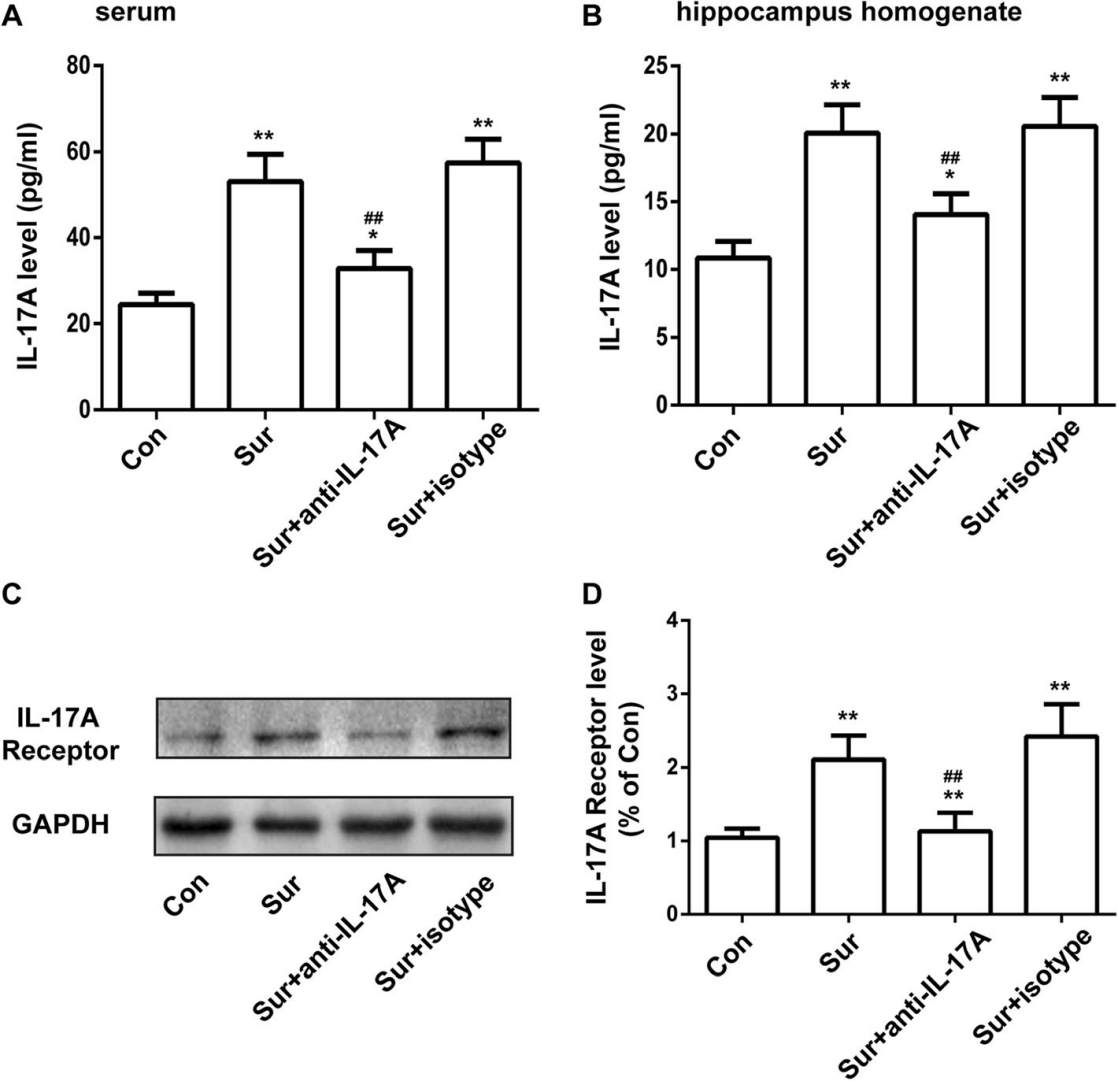
Tibial fracture surgery increased serum and hippocampus IL-17A levels and hippocampus IL-17A receptor expression. The level of IL-17A in serum (**a**) and the hippocampus (**b**) 24 h after surgery was determined by ELISA. The expression of IL-17A receptors in the hippocampus 24 h after surgery was detected by Western blot (**c**). The expression of IL-17A receptors was quantified and normalized to GAPDH levels (**d**). *P < 0.05 and **P < 0.01 versus the Con group. ^##^*P* < 0.01 versus the Sur group. Data are presented as the mean ± SEM (*n* = 6)

The level of IL-17A in serum and the hippocampus 24 h after surgery was quantified by ELISA. As shown in Fig. 3a, b, tibial fracture surgery increased the level of IL-17A in serum and the hippocampus compared to controls, which was reversed by treatment with anti-IL-17A Abs. At the same time, we detected the expression of IL-17A receptors in the hippocampus by Western blot. The expression of IL-17A receptors in the hippocampus was also increased after surgery compared with controls, which was reversed by treatment with anti-IL-17A Abs (Fig. 3c, d). Treatment with the isotype control had no effect on the level of IL-17A or IL-17A receptors.

### Anti-IL-17A Abs attenuated the surgery-induced expression of IL-6 and IL-1β in the hippocampus

**Fig. 4:**
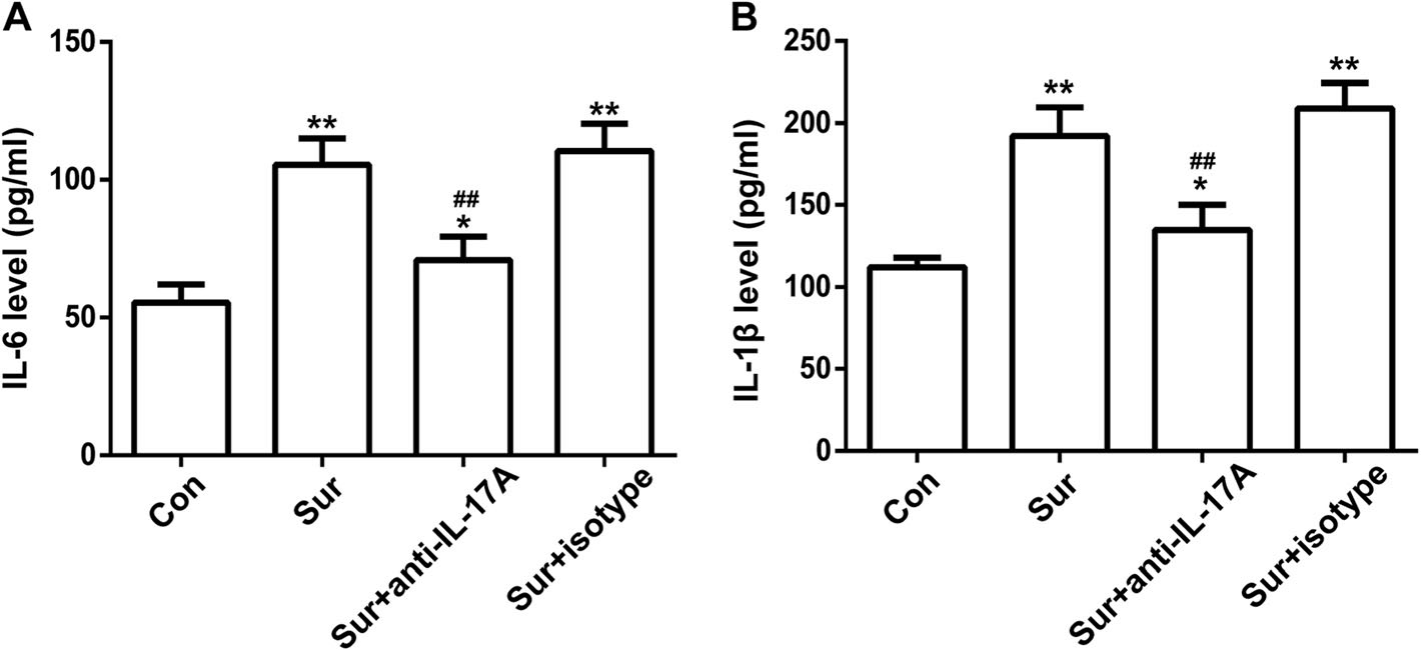
Anti-IL-17A Abs attenuated the surgery-induced expression of IL-6 and IL-1β in the hippocampus. The level of IL-6 (**a**) and IL-1β (**b**) in the hippocampus 24 h after surgery was determined by ELISA. *P < 0.05 and **P < 0.01 versus the Con group. ^##^*P* < 0.01 versus the Sur group. Data are presented as the mean ± SEM (*n* = 6)

Since neuroinflammation plays a critical role in the pathophysiology of PND, to examine whether anti-IL-17A Abs could suppress surgery-induced neuroinflammation in aged mice, we tested the expression levels of IL-6 and IL-1β in the hippocampus 24 h after surgery by ELISA. As shown in Fig. 4, surgery significantly increased IL-6 and IL-1β content in the hippocampus compared to the control. However, injection with anti-IL-17A Abs 30 min prior to surgery attenuated the surgery-induced increased level of IL-6 and IL-1β. Injection with an isotype control had no effect on the expression of IL-6 and IL-1β. These results suggested injection with anti-IL-17A Abs could alleviate the production of pro-inflammatory factors.

### Anti-IL-17A Abs reduced the surgery-induced EB leakage and expression of albumin in the hippocampus

**Fig. 5:**
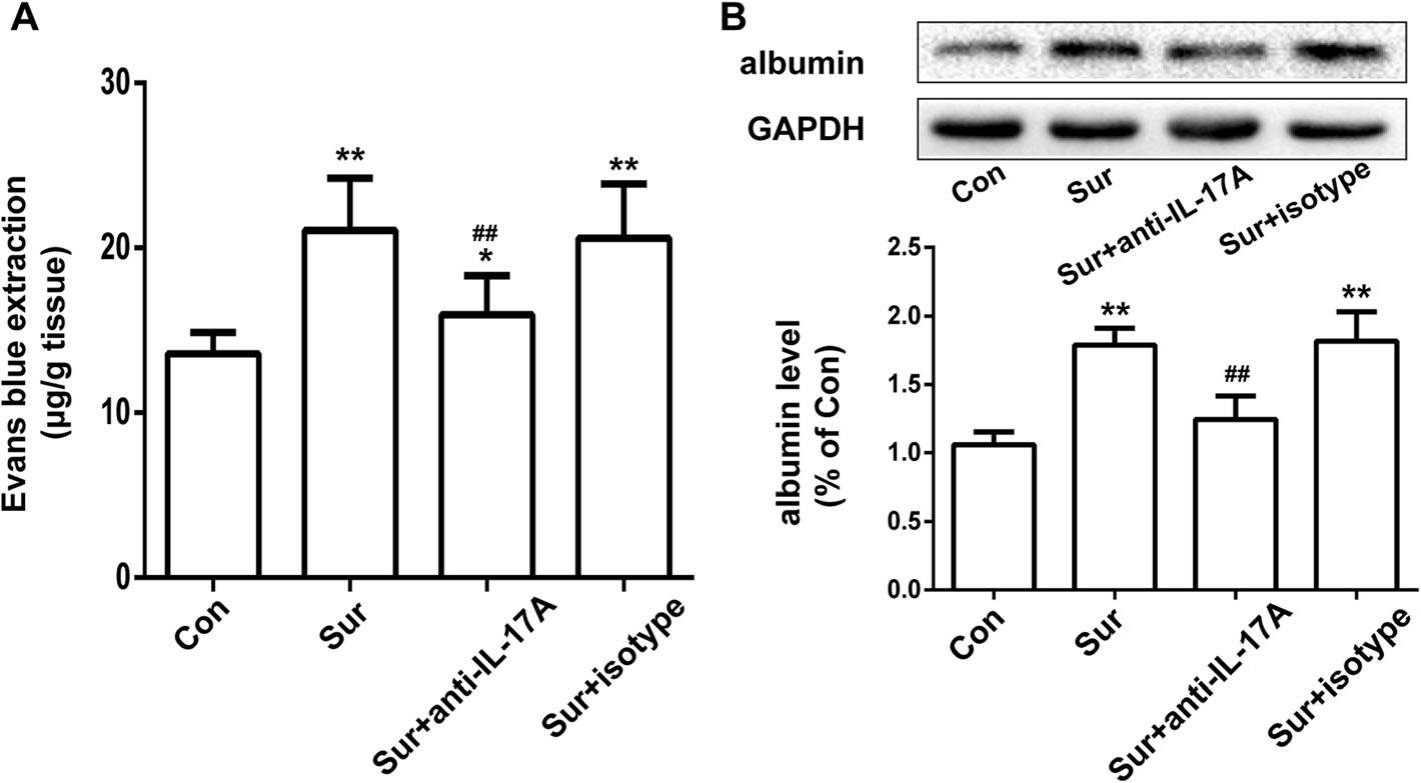
Anti-IL-17A Abs reduced the surgery-induced EB leakage and expression of albumin in the hippocampus. The EB leakage (**a**) and the albumin content (**b**) tested by Western blot in the hippocampus 24 h after surgery were used to determine BBB integrity. *P < 0.05 and **P < 0.01 versus the Con group. ^##^*P* < 0.01 versus the Sur group. Data are presented as mean ± SEM (*n* = 4)

To further explore whether tibial fracture surgery could disrupt the integrity of the BBB and the modulatory effect of IL-17A, we detected leakage of EB in the hippocampus. Compared with the control group, the EB content in the hippocampus was notably increased 24 h after surgery. However, pretreatment with anti-IL-17A Abs reduced the surgery-induced EB leakage in the hippocampus (Fig. 5a). To further confirm the impact on the integrity of the BBB, we tested the albumin content in the hippocampus by Western blot. Pretreatment with anti-IL-17A Abs attenuated the surgery-induced increase in albumin content in the hippocampus (Fig. 5b). Pretreatment with the isotype control had no effect on the level of EB leakage and albumin. These results suggested that pretreatment with anti-IL-17A Abs reduced the surgery-induced BBB integrity disruption.

### Anti-IL-17A Abs reversed the surgery-induced decrease of occludin and claudin-5 levels in the hippocampus

**Fig. 6:**
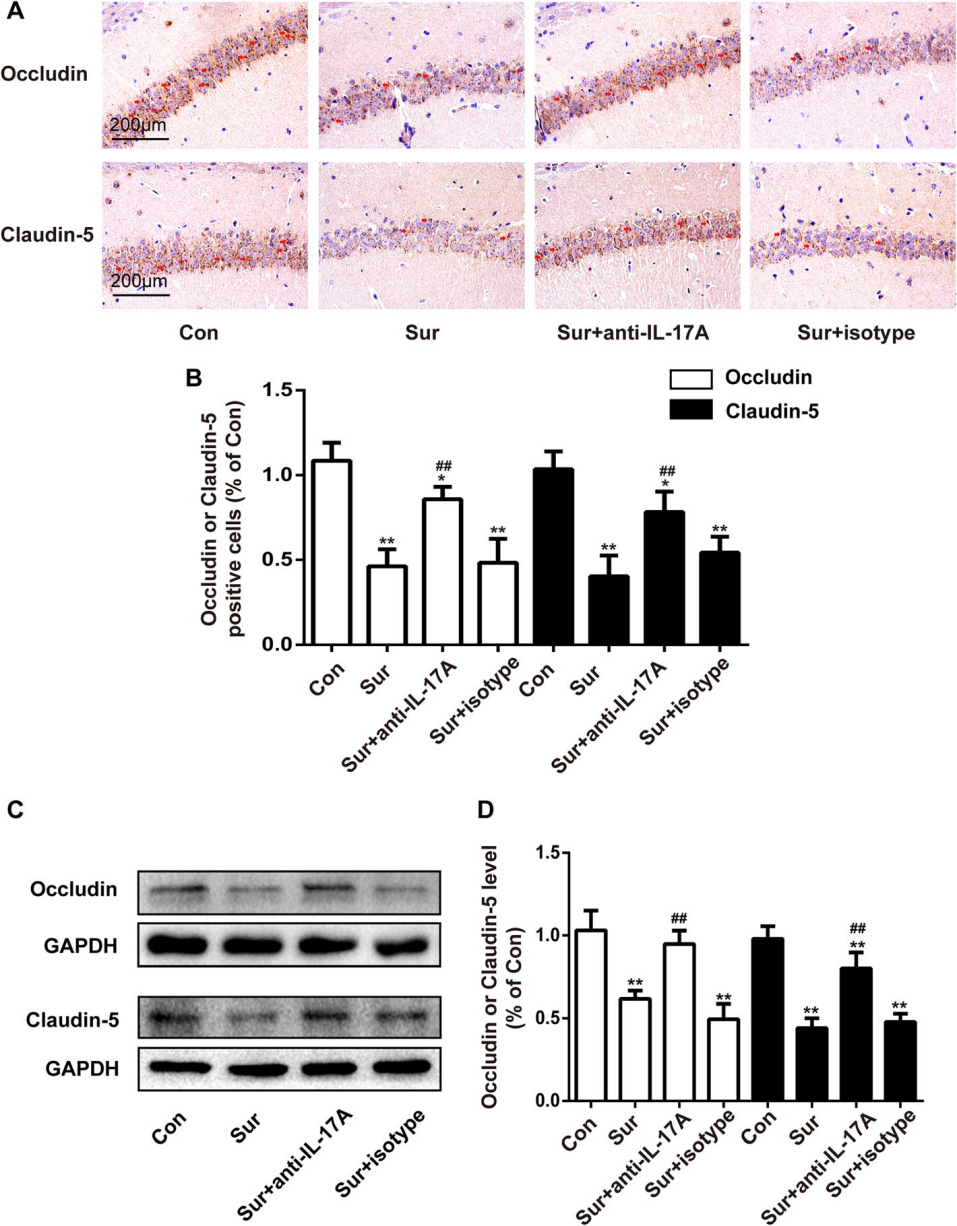
Anti-IL-17A Abs reversed the surgery-induced decrease of occludin and claudin-5 levels in the hippocampus. **a** The expression levels of occludin and claudin-5 in the hippocampus detected by immunostaining. Scale bar, 200 μm. **b** Quantification of occludin-positive and claudin-positive cells in the CA1 area of the hippocampus. **c** The expression levels of occludin and claudin-5 in the hippocampus detected by Western blot. **d** Quantification of occludin and claudin-5 levels. *P < 0.05 and **P < 0.01 versus the Con group. ^##^*P* < 0.01 versus the Sur group. Data are presented as the mean ± SEM (*n* = 4)

TJs, mainly occludin and claudin-5, are crucial factors responsible for BBB integrity. To further explore the impact on BBB integrity, we evaluated the level of occludin and claudin-5 by immunohistochemistry staining and Western blot. As shown in Fig. 6, surgery for tibial fracture induced a decrease in occludin and claudin-5 levels, which was reversed by pretreatment with anti-IL-17A Abs. Pretreatment with the isotype control had no effect on the levels of occludin and claudin-5 in that hippocampus. These results suggested that injection with anti-IL-17A Abs inhibited the surgery-induced reduction in occludin and claudin-5 levels.

### Anti-IL-17A Abs inhibited the expression of MMP-2 and MMP-9 induced by surgery

**Fig. 7:**
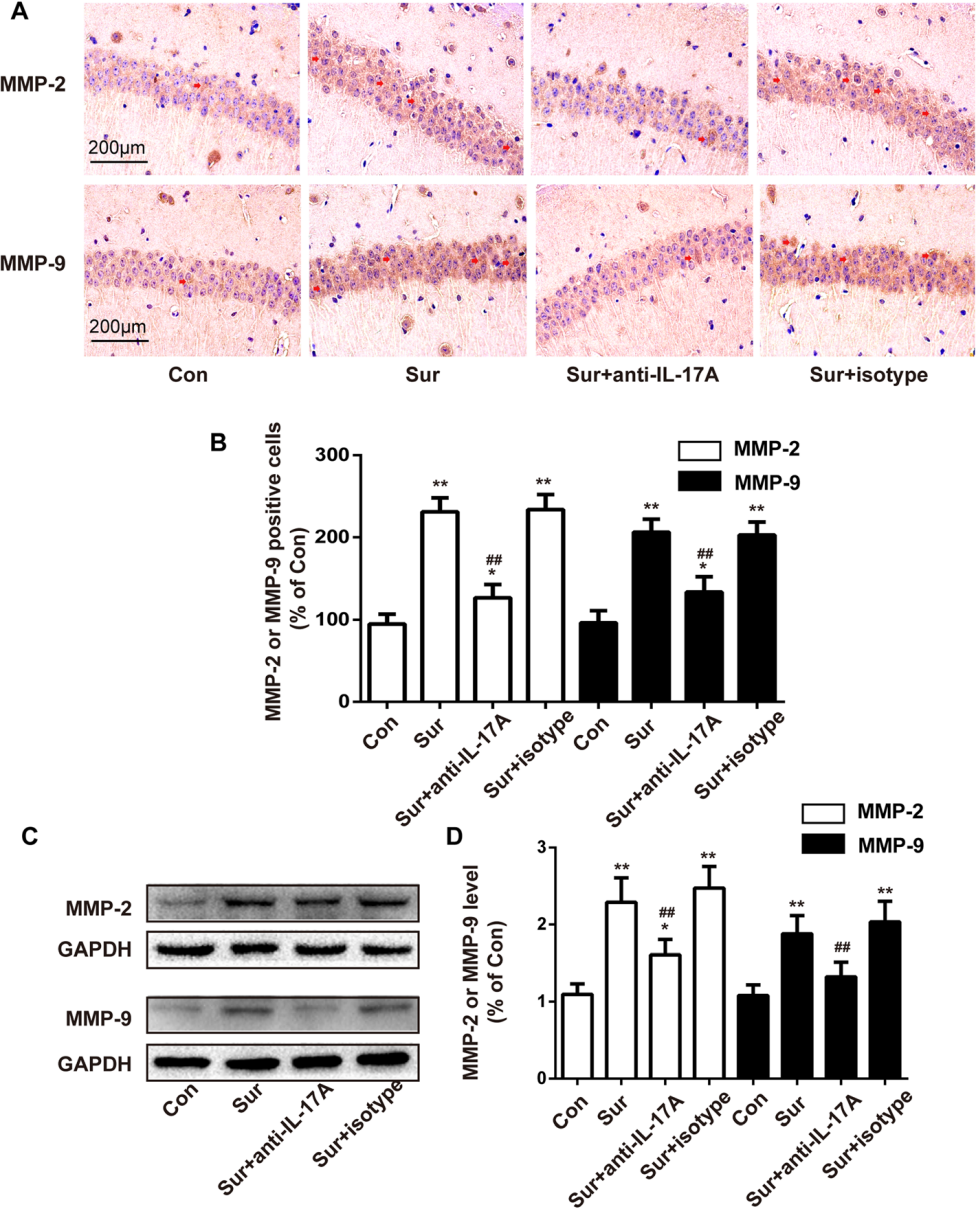
Anti-IL-17A Abs inhibited the expression of MMP-2 and MMP-9 induced by surgery. **a** Immunostaining was used to detect the levels of MMP-2 and MMP-9 in the CA1 area of the hippocampus. Scale bar, 200 μm. **b** Quantification of MMP-2-positive and MMP-9-positive cells in the CA1 area of the hippocampus. **c** The expression levels of MMP-2 and MMP-9 in the hippocampus detected by Western blot. **d** Quantification of MMP-2 and MMP-9 levels. *P < 0.05 and **P < 0.01 versus the Con group. ^##^*P* < 0.01 versus the Sur group. Data are presented as the mean ± SEM (*n* = 4)

MMPs, mainly MMP-2 and MMP-9, are vital components of the extracellular matrix proteasome, and contribute to BBB leakage by disrupting the neurovascular matrix and degrading occluding and claudin-5. Accordingly, we evaluated the levels of MMP-2 and MMP-9 in the hippocampus by immunohistochemistry staining and Western blot. As shown in Fig. 7, upon surgical treatment, MMP-2 and MMP-9 levels in the hippocampus of mice who underwent surgery were significantly higher than the levels in control mice, but these elevations were notably inhibited by anti-IL-17A Abs. Injection with the isotype control had no effect on the levels of MMP-2 or MMP-9 in the hippocampus. Full blots for Figs. 6 and 7 were included in Additional file 2. These results suggested that injection with anti-IL-17A Abs reversed the surgery-induced increased levels of MMP-2 and MMP-9.

### IL-17A upregulated the expression of IL-17A receptor in bEnd.3 cells

**Fig. 8:**
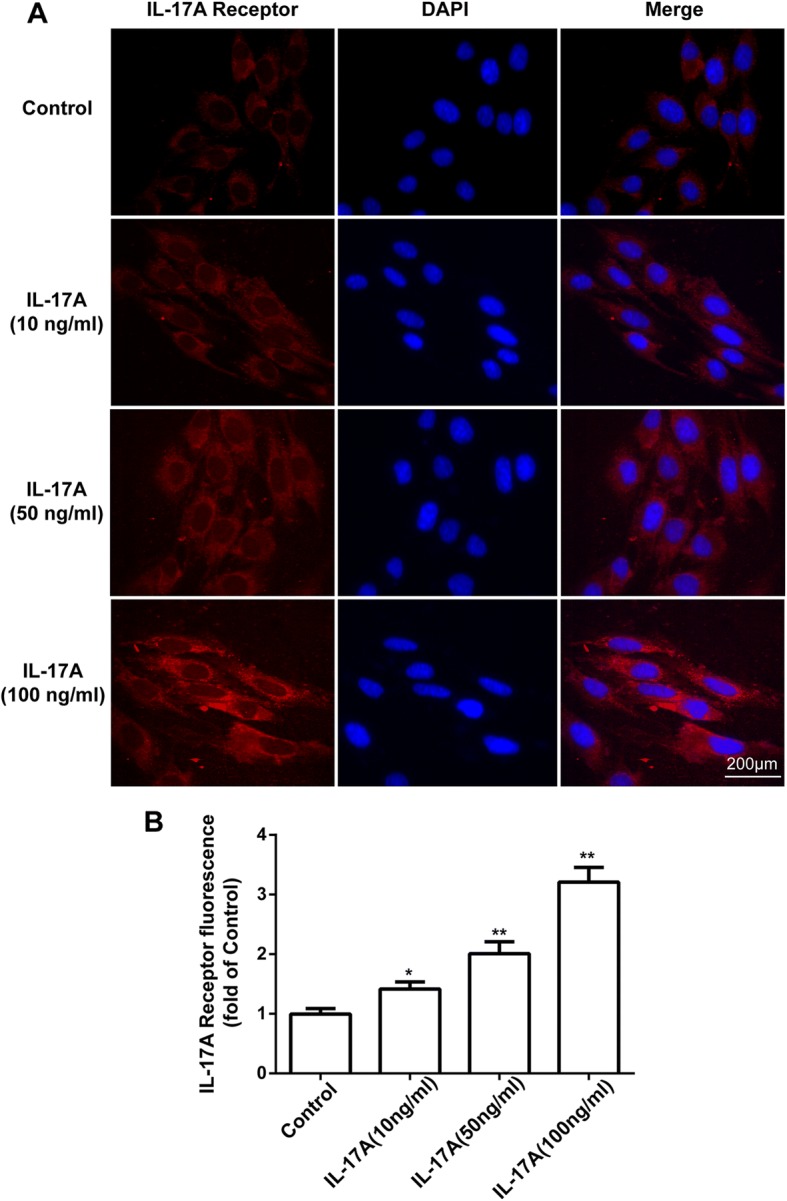
IL-17A upregulated the expression of IL-17A receptor in bEnd.3 cells. **a** Cells were stained with IL-17A receptor antibodies (red). IL-17A receptor expression in bEnd.3 cells was observed using confocal scanning. Blue staining represents DAPI. Scale bar, 200 μm. **b** Quantitative data of the mean intensity of IL-17A receptor fluorescence in bEnd.3 cells. All experiments were repeated three times. *P < 0.05 and **P < 0.01 versus the control group. Data are presented as the mean ± SEM

To ascertain whether IL-17A modulates the expression of IL-17A receptor proteins in bEnd.3 cells, immunofluorescence was used in the present study. As shown in Fig. 8, after incubation with different concentrations of IL-17A (10, 50 and 100 ng/ml) for 24 h, the expression of IL-17A receptors (in red) was greatly upregulated in bEnd.3 cells. These results suggest that IL-17A could upregulate the expression of IL-17A receptor in bEnd.3 cells.

### IL-17A decreased occludin and claudin-5 levels and increased MMP-2 and MMP-9 levels in bEnd.3 cells

**Fig. 9:**
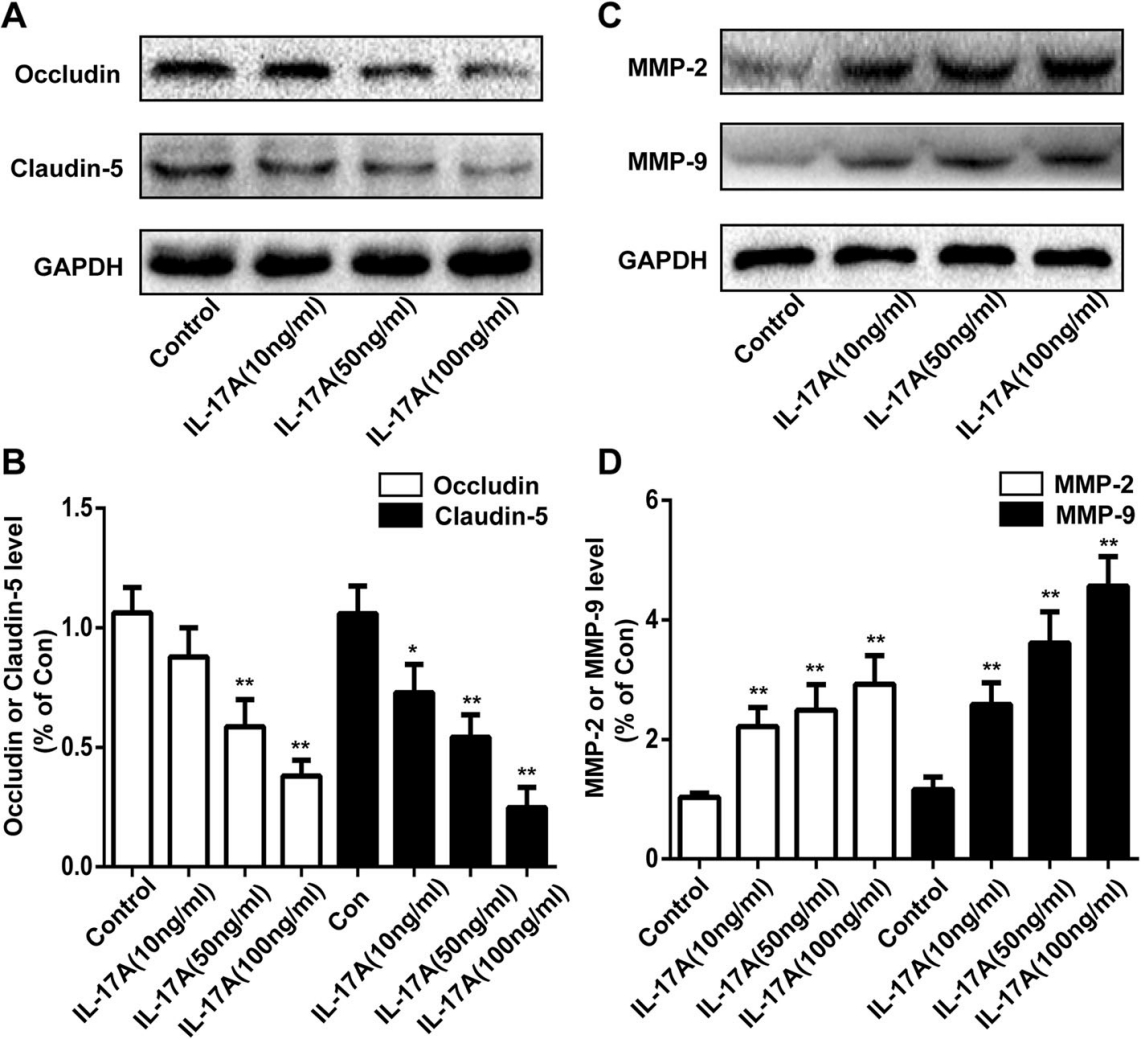
IL-17A decreased occludin and claudin-5 levels and increased MMP-2 and MMP-9 levels in bEnd.3 cells. **a** The expression levels of occludin and claudin-5 in bEnd.3 cells 24 h after treatment with different doses of IL-17A detected by Western blot. **b** Quantification of occludin and claudin-5 in bEnd.3 cells. **c** The expression levels of MMP-2 and MMP-9 in bEnd.3 cells 24 h after treatment with different doses of IL-17A detected by Western blot. **d** Quantification of MMP-2 and MMP-9 levels in bEnd.3 cells. All experiments were repeated three times. *P < 0.05 and **P < 0.01 versus the control group. Data are presented as the mean ± SEM

To further investigate how IL-17A disrupted BBB integrity, a cell culture model of bEnd.3 cells was used. bEnd.3 cells were cultured with different doses of IL-17A (10, 50 and 100 ng/ml) for 24 h. We examined the expression of TJs and MMPs by Western blot. As shown in Fig. 9, IL-17A treatment at concentrations of 50 ng/ml or greater for 24 h caused decreased expression of occludin and at concentrations of 10 ng/ml or greater caused claudin-5 decrease in bEnd.3 cells. Meanwhile, the MMP-2 and MMP-9 levels were increased at concentrations of 10 ng/ml or greater in bEnd.3 cells. These observations suggested that IL-17A might induce BBB disruption by decreasing occludin and claudin-5 protein expression levels and increasing levels of MMP-2 and MMP-9 proteins.

## Discussion

There is a close connection between IL-17A expression and the pathogenesis of CNS inflammatory diseases, including depression, ischaemic brain injury and multiple sclerosis [31–33]. However, it is not clear whether IL-17A is involved in the development of PND. In this study, we investigated the impact of IL-17A on cognitive change, neuroinflammation and BBB function after surgery. We found that surgery induced cognitive decline, elevation of IL-17A and IL-17A receptor levels, production of pro-inflammatory cytokines and BBB dysfunction. Treatment with anti-IL-17A Abs inhibited the surgery-induced neuroinflammation and cognitive decline via alleviating the BBB disruption.

Research has suggested that surgery could impair learning and memory abilities, especially in aged rodents. It manifests as deficits in exploratory behaviour and spatially based working memory [34]. Contextual fear conditioning and Y maze tests are widely used to assess learning and memory abilities in rodents [35, 36]. Our results demonstrated that the freezing behaviour of mice decreased following surgery, whereas pretreatment with anti-IL-17A Abs could partially reverse the decrease in freezing time. In accordance with the contextual fear conditioning results, surgery increased the number of learning trials, which could be prevented by pretreatment with anti-IL-17A Abs. Taken together, our study indicated that pretreatment with anti-IL-17A Abs could inhibit cognitive deficits following surgery, indicating that IL-17A was involved in surgery-induced cognitive decline.Neuroinflammation plays an important role in many neurodegenerative diseases, including Alzheimer’s disease, Parkinson’s disease and depression [37–39], and is believed to be a vital pathological mechanism of postoperative cognitive dysfunction [40]. Levels of pro-inflammatory cytokines and markers have been shown to contribute to cognitive dysfunction and increase with age [41–43]. Surgery-caused increases of plasma cytokines are associated with the expression of cytokines in the hippocampus and memory impairment. Functional inhibition of IL-1β could mitigate the neuroinflammation, and peripheral TNF-α blockade could reduce the release of IL-1β and prevent neuroinflammation and postoperative cognitive decline [44, 45]. During CNS inflammation, IL-17A could also stimulate the production of pro-inflammatory cytokines and chemokines in CNS-resident cells and mediate leukocyte recruitment [46, 47]. Our results suggested that the level of IL-17A in serum and hippocampus, along with IL-6 and IL-1β in the hippocampus, increased 24 h after surgery compared with controls, and pretreatment with anti-IL-17A Abs abolished the increase in IL-6 and IL-1β following surgery. This in vivo experiment indicated that IL-17A played a role in surgery-induced neuroinflammation.

BBB disruption could precede neuroinflammation and cognitive dysfunction [48, 49]. And the immunity plays an important role in maintaining the function of the blood-brain barrier. Peripheral surgery may disrupt the BBB via complement activation and pro-inflammatory cytokines, such as TNF-α and high mobility group box-1 (HMGB1), which promotes brain inflammation [50, 51]. Furthermore, BBB disruption facilitates the migration of macrophages into the brain, and the hippocampal recruitment of macrophages is necessary for neuroinflammation and memory dysfunction, inducing cognitive decline following surgery [52, 53].

Some research has indicated that IL-17A decreased the integrity of the BBB, which contributes to the development of EAE [31]. To unravel whether IL-17A contributed to neuroinflammation and cognitive dysfunction after surgery via impairing BBB integrity, we performed in vivo and in vitro experiments. Hania Kebir demonstrated that human CNS postmortem materials from heavily inflamed MS individuals showed more IL-17R expression on the endothelium compared with unaffected control individuals [54]. We found that expression of IL-17A receptors was elevated following surgery, and IL-17A could increase the expression of IL-17A receptors in bEnd.3 cells. To evaluate the effect of surgery on BBB integrity and the role of IL-17A in the process, we tested EB extravasation and expression of albumin in the hippocampus and found obviously elevated EB extravasation and the expression of albumin 24 h after surgery, which was prevented by pretreatment with anti-IL-17A Abs. These data suggested that IL-17A is involved in surgery-induced disruption of BBB integrity.

Endothelial cells of the BBB are different from endothelial cells in other tissues because of continuous intercellular TJs, which are associated with BBB permeability and involved in many CNS diseases [55, 56]. The occludin and claudin families are important components of TJs [57]. Our study demonstrated that surgery reduced the levels of occludin and claudin-5 in the hippocampus, which was inhibited by pretreatment with anti-IL-17A Abs. MMPs, a family of proteolytic enzymes, can break down the extracellular matrix proteins of the basement membranes and degrade the TJs [58]. The involvement of MMP-2 and MMP-9 in BBB disruption has been reported in several ischaemic injury studies [59, 60]. In our study, expressions of MMP-2 and MMP-9 were significantly increased after surgery, which was obviously inhibited by pretreatment with anti-IL-17A Abs. Furthermore, our in vitro experiment suggested that IL-17A could decrease the expression of occludin and claudin-5 and increase the expression of MMP-2 and MMP-9 in bEnd.3 cells. These data further suggested that IL-17A is involved in surgery-induced disruption of BBB permeability.

## Conclusions

In conclusion, our study revealed that IL-17A was involved in neuroinflammation and cognitive dysfunction after surgery in aged mice via BBB disruption. The in vitro study further confirmed that IL-17A could upregulate IL-17A receptor expression in bEnd.3 cells, increase MMP levels and decrease TJs, which disrupted BBB integrity. Our results might provide not only a better understanding of the role of IL-17A in PND but also a new therapeutic strategy for neuroinflammation-related diseases.

## Additional files

Additional file 1: Training and learning data. Table A. Freezing time before shock during the training period. Table B. Freezing time after shock 1 during the training period. Table C. Freezing time after shock 2 during the training period. Table D. Freezing time in contextual fear test. (*n* = 12). (PDF 73 kb)

Additional file 2: Original full Western blotting images for Figs. 6 and 7. The cross-section was not included in this study. (PDF 4281 kb)

## Abbreviations

Abs: Neutralizing antibody
AD: Alzheimer’s disease
BBB: Blood-brain barrier
CNS: Central nervous system
DAPI: 4,6-Diamidino-2phenylindole
DMEM: Dulbecco’s modified Eagle’s medium
EAE: Experimental autoimmune encephalomyelitis
EB: Evans blue
FBS: Foetal bovine serum
IL-17A: Interleukin-17A
MMP-2: Matrix metalloproteinase-2
MMP-9: Matrix metalloproteinase-9
MS: Multiple sclerosis
PD: Parkinson’s disease
PMSF: Phenylmethylsulfonylfluoride
PND: Perioperative neurocognitive disorders
SDS-PAGE: Sodium dodecyl sulfate-polyacrylamide gel electrophoresis
TFC: Trace fear conditioning
TJs: Tight junctions

## References

[1] Evered L, Silbert B, Knopman DS, Scott DA, Dekosky ST, Rasmussen LS, Oh ES, Crosby G, Berger M, Eckenhoff RG (2018). Recommendations for the nomenclature of cognitive change associated with anaesthesia and surgery-2018. Br J Anaesth.

[2] Hovens IB, Schoemaker RG, van der Zee EA, Heineman E, Izaks GJ, van Leeuwen BL (2012). Thinking through postoperative cognitive dysfunction: how to bridge the gap between clinical and pre-clinical perspectives. Brain Behav Immun.

[3] Shoair OA, Grasso Ii MP, Lahaye LA, Daniel R, Biddle CJ, Slattum PW (2015). Incidence and risk factors for postoperative cognitive dysfunction in older adults undergoing major noncardiac surgery: a prospective study. J Anaesthesiol Clin Pharmacol.

[4] Hovens IB, Schoemaker RG, van der Zee EA, Absalom AR, Heineman E, van Leeuwen BL (2014). Postoperative cognitive dysfunction: involvement of neuroinflammation and neuronal functioning. Brain Behav Immun.

[5] Vacas S, Degos V, Feng X, Maze M (2013). The neuroinflammatory response of postoperative cognitive decline. Br Med Bull.

[6] Li X, Robertson CM, Yu X, Cheypesh A, Dinu IA, Li J (2014). Early postoperative systemic inflammatory response is an important determinant for adverse 2-year neurodevelopment-associated outcomes after the Norwood procedure. J Thorac Cardiovasc Surg.

[7] Skvarc DR, Berk M, Byrne LK, Dean OM, Dodd S, Lewis M, Marriott A, Moore EM, Morris G, Page RS, Gray L (2018). Post-operative cognitive dysfunction: an exploration of the inflammatory hypothesis and novel therapies. Neurosci Biobehav Rev.

[8] Christensen LB, Woods TA, Carmody AB, Caughey B, Peterson KE (2014). Age-related differences in neuroinflammatory responses associated with a distinct profile of regulatory markers on neonatal microglia. J Neuroinflammation.

[9] Cortese GP, Barrientos RM, Maier SF, Patterson SL (2011). Aging and a peripheral immune challenge interact to reduce mature brain-derived neurotrophic factor and activation of TrkB, PLCgamma1, and ERK in hippocampal synaptoneurosomes. J Neurosci.

[10] Sweeney MD, Sagare AP, Zlokovic BV (2018). Zlokovic, blood-brain barrier breakdown in Alzheimer disease and other neurodegenerative disorders. Nat Rev Neurol.

[11] Zlokovic BV (2008). The blood-brain barrier in health and chronic neurodegenerative disorders. Neuron.

[12] Friese MA, Schattling B, Fugger L (2014). Mechanisms of neurodegeneration and axonal dysfunction in multiple sclerosis. Nat Rev Neurol.

[13] Cabezas R, Avila M, Gonzalez J, El-Bacha RS, Baez E, Garcia-Segura LM, Jurado Coronel JC, Capani F, Cardona-Gomez GP, Barreto GE (2014). Astrocytic modulation of blood brain barrier: perspectives on Parkinson’s disease. Front Cell Neurosci.

[14] Montagne A, Barnes SR, Sweeney MD, Halliday MR, Sagare AP, Zhao Z, Toga AW, Jacobs RE, Liu CY, Amezcua L, Harrington MG, Chui HC, Law M, Zlokovic BV (2015). Blood-brain barrier breakdown in the aging human hippocampus. Neuron.

[15] Gorle N, Van Cauwenberghe C, Libert C, Vandenbroucke RE (2016). The effect of aging on brain barriers and the consequences for Alzheimer’s disease development. Mamm Genome.

[16] Merino JG, Latour LL, Tso A, Lee KY, Kang DW, Davis LA, Lazar RM, Horvath KA, Corso PJ, Warach S (2013). Blood-brain barrier disruption after cardiac surgery. AJNR Am J Neuroradiol.

[17] Varvel NH, Neher JJ, Bosch A, Wang W, Ransohoff RM, Miller RJ, Dingledine R (2016). Infiltrating monocytes promote brain inflammation and exacerbate neuronal damage after status epilepticus. Proc Natl Acad Sci U S A.

[18] Becher B, Segal BM (2011). T (H)17 cytokines in autoimmune neuro-inflammation. Curr Opin Immunol.

[19] Waisman A, Hauptmann J, Regen T (2015). The role of IL-17 in CNS diseases. Acta Neuropathol.

[20] Reinhold D, Bank U, Tager M, Ansorge S, Wrenger S, Thielitz A, Lendeckel U, Faust J, Neubert K, Brocke S (2008). DP IV/CD26, APN/CD13 and related enzymes as regulators of T cell immunity: implications for experimental encephalomyelitis and multiple sclerosis. Front Biosci.

[21] Zhang X, Tao Y, Troiani L, Markovic-Plese S (2011). Simvastatin inhibits IFN regulatory factor 4 expression and Th17 cell differentiation in CD4+ T cells derived from patients with multiple sclerosis. J Immunol.

[22] Sun J, Zhang S, Zhang X, Zhang X, Dong H, Qian Y (2015). IL-17A is implicated in lipopolysaccharide-induced neuroinflammation and cognitive impairment in aged rats via microglial activation. J Neuroinflammation.

[23] Wang G, Wu K, Li W, Zhao E, Shi L, Wang J, Shuai X, Cai K, Lu X, Tao K, Wang G (2014). Role of IL-17 and TGF-beta in peritoneal adhesion formation after surgical trauma. Wound Repair Regen.

[24] Huppert J, Closhen D, Croxford A, White R, Kulig P, Pietrowski E, Bechmann I, Becher B, Luhmann HJ, Waisman A, Kuhlmann CR (2010). Cellular mechanisms of IL-17-induced blood-brain barrier disruption. FASEB J.

[25] Baeten D, Sieper J, Braun J, Baraliakos X, Dougados M, Emery P, Deodhar A, Porter B, Martin R, Andersson M, Mpofu S, Richards HB (2015). Secukinumab, an interleukin-17A inhibitor, in ankylosing spondylitis. N Engl J Med.

[26] Reich K, Papp KA, Matheson RT, Tu JH, Bissonnette R, Bourcier M, Gratton D, Kunynetz RA, Poulin Y, Rosoph LA, Stingl G, Bauer WM, Salter JM, Falk TM, Blodorn-Schlicht NA, Hueber W, Sommer U, Schumacher MM, Peters T, Kriehuber E, Lee DM, Wieczorek GA, Kolbinger F, Bleul CC (2015). Evidence that a neutrophil-keratinocyte crosstalk is an early target of IL-17A inhibition in psoriasis. Exp Dermatol.

[27] Genovese MC, Greenwald M, Cho CS, Berman A, Jin L, Cameron GS, Benichou O, Xie L, Braun D, Berclaz PY, Banerjee S (2014). A phase II randomized study of subcutaneous ixekizumab, an anti-interleukin-17 monoclonal antibody, in rheumatoid arthritis patients who were naive to biologic agents or had an inadequate response to tumor necrosis factor inhibitors. Arthritis Rheumatol.

[28] Sommer A, Maxreiter F, Krach F, Fadler T, Grosch J, Maroni M, Graef D, Eberhardt E, Riemenschneider MJ, Yeo GW, Kohl Z, Xiang W, Gage FH, Winkler J, Prots I, Winner B (2018). Th17 lymphocytes induce neuronal cell death in a human iPSC-based model of Parkinson’s disease. Cell Stem Cell.

[29] Zhang X, Yao H, Qian Q, Li N, Jin W, Qian Y (2016). Cerebral mast cells participate in postoperative cognitive dysfunction by promoting astrocyte activation. Cell Physiol Biochem.

[30] Xiong C, Zhang Z, Baht GS, Terrando N. A mouse model of orthopedic surgery to study postoperative cognitive dysfunction and tissue regeneration. JoVE-J Vis Exp. 2018:132. 10.3791/56701 PMC5916114 29553500

[31] Nadeem A, Ahmad SF, Al-Harbi NO, Fardan AS, El-Sherbeeny AM, Ibrahim KE, Attia SM (2017). IL-17A causes depression-like symptoms via NFkappaB and p38MAPK signaling pathways in mice: implications for psoriasis associated depression. Cytokine.

[32] Gelderblom M, Weymar A, Bernreuther C, Velden J, Arunachalam P, Steinbach K, Orthey E, Arumugam TV, Leypoldt F, Simova O, Thom V, Friese MA, Prinz I, Holscher C, Glatzel M, Korn T, Gerloff C, Tolosa E, Magnus T (2012). Neutralization of the IL-17 axis diminishes neutrophil invasion and protects from ischemic stroke. Blood.

[33] Kostic M, Dzopalic T, Zivanovic S, Zivkovic N, Cvetanovic A, Stojanovic I, Vojinovic S, Marjanovic G, Savic V, Colic M (2014). IL-17 and glutamate excitotoxicity in the pathogenesis of multiple sclerosis. Scand J Immunol.

[34] Barrientos RM, Hein AM, Frank MG, Watkins LR, Maier SF (2012). Intracisternal interleukin-1 receptor antagonist prevents postoperative cognitive decline and neuroinflammatory response in aged rats. J Neurosci.

[35] Feng X, Degos V, Koch LG, Britton SL, Zhu Y, Vacas S, Terrando N, Nelson J, Su X, Maze M (2013). Surgery results in exaggerated and persistent cognitive decline in a rat model of the metabolic syndrome. Anesthesiology.

[36] Webster SJ, Bachstetter AD, Nelson PT, Schmitt FA, Van Eldik LJ (2014). Using mice to model Alzheimer’s dementia: an overview of the clinical disease and the preclinical behavioral changes in 10 mouse models. Front Genet.

[37] Heneka MT, Carson MJ, El KJ, Landreth GE, Brosseron F, Feinstein DL, Jacobs AH, Wyss-Coray T, Vitorica J, Ransohoff RM, Herrup K, Frautschy SA, Finsen B, Brown GC, Verkhratsky A, Yamanaka K, Koistinaho J, Latz E, Halle A, Petzold GC, Town T, Morgan D, Shinohara ML, Perry VH, Holmes C, Bazan NG, Brooks DJ, Hunot S, Joseph B, Deigendesch N, Garaschuk O, Boddeke E, Dinarello CA, Breitner JC, Cole GM, Golenbock DT, Kummer MP (2015). Neuroinflammation in Alzheimer’s disease. Lancet Neurol.

[38] De Virgilio A, Greco A, Fabbrini G, Inghilleri M, Rizzo MI, Gallo A, Conte M, Rosato C, Ciniglio AM, de Vincentiis M (2016). Parkinson’s disease: autoimmunity and neuroinflammation. Autoimmun Rev.

[39] Bhattacharya A, Drevets WC (2017). Role of neuro-immunological factors in the pathophysiology of mood disorders: implications for novel therapeutics for treatment resistant depression. Curr Top Behav Neurosci.

[40] Kapila AK, Watts HR, Wang T, Ma D (2014). The impact of surgery and anesthesia on post-operative cognitive decline and Alzheimer’s disease development: biomarkers and preventive strategies. J Alzheimers Dis.

[41] Trapero I, Cauli O (2014). Interleukin 6 and cognitive dysfunction. Metab Brain Dis.

[42] Cho SH, Chen JA, Sayed F, Ward ME, Gao F, Nguyen TA, Krabbe G, Sohn PD, Lo I, Minami S, Devidze N, Zhou Y, Coppola G, Gan L (2015). SIRT1 deficiency in microglia contributes to cognitive decline in aging and neurodegeneration via epigenetic regulation of IL-1beta. J Neurosci.

[43] Michaud M, Balardy L, Moulis G, Gaudin C, Peyrot C, Vellas B, Cesari M, Nourhashemi F (2013). Proinflammatory cytokines, aging, and age-related diseases. J Am Med Dir Assoc.

[44] Terrando N, Monaco C, Ma D, Foxwell BM, Feldmann M, Maze M (2010). Tumor necrosis factor-alpha triggers a cytokine cascade yielding postoperative cognitive decline. Proc Natl Acad Sci U S A.

[45] Cibelli M, Fidalgo AR, Terrando N, Ma D, Monaco C, Feldmann M, Takata M, Lever IJ, Nanchahal J, Fanselow MS, Maze M (2010). Role of interleukin-1beta in postoperative cognitive dysfunction. Ann Neurol.

[46] Zepp J, Wu L, Li X (2011). IL-17 receptor signaling and T helper 17-mediated autoimmune demyelinating disease. Trends Immunol.

[47] Xiao Y, Jin J, Chang M, Nakaya M, Hu H, Zou Q, Zhou X, Brittain GC, Cheng X, Sun SC (2014). TPL2 mediates autoimmune inflammation through activation of the TAK1 axis of IL-17 signaling. J Exp Med.

[48] Obermeier B, Verma A, Ransohoff RM (2016). The blood-brain barrier. Handb Clin Neurol.

[49] Yang S, Gu C, Mandeville ET, Dong Y, Esposito E, Zhang Y, Yang G, Shen Y, Fu X, Lo EH, Xie Z (2017). Anesthesia and surgery impair blood-brain barrier and cognitive function in mice. Front Immunol.

[50] He HJ, Wang Y, Le Y, Duan KM, Yan XB, Liao Q, Liao Y, Tong JB, Terrando N, Ouyang W (2012). Surgery upregulates high mobility group box-1 and disrupts the blood-brain barrier causing cognitive dysfunction in aged rats. CNS Neurosci Ther.

[51] Xiong C, Liu J, Lin D, Zhang J, Terrando N, Wu A (2018). Complement activation contributes to perioperative neurocognitive disorders in mice. J Neuroinflammation.

[52] Terrando N, Eriksson LI, Ryu JK, Yang T, Monaco C, Feldmann M, Jonsson FM, Charo IF, Akassoglou K, Maze M (2011). Resolving postoperative neuroinflammation and cognitive decline. Ann Neurol.

[53] Degos V, Vacas S, Han Z, van Rooijen N, Gressens P, Su H, Young WL, Maze M (2013). Depletion of bone marrow-derived macrophages perturbs the innate immune response to surgery and reduces postoperative memory dysfunction. Anesthesiology.

[54] Kebir H, Kreymborg K, Ifergan I, Dodelet-Devillers A, Cayrol R, Bernard M, Giuliani F, Arbour N, Becher B, Prat A (2007). Human TH17 lymphocytes promote blood-brain barrier disruption and central nervous system inflammation. Nat Med.

[55] Abbott NJ, Ronnback L, Hansson E (2006). Astrocyte-endothelial interactions at the blood-brain barrier. Nat Rev Neurosci.

[56] Obermeier B, Daneman R, Ransohoff RM (2013). Development, maintenance and disruption of the blood-brain barrier. Nat Med.

[57] Fontijn RD, Rohlena J, van Marle J, Pannekoek H, Horrevoets AJ (2006). Limited contribution of claudin-5-dependent tight junction strands to endothelial barrier function. Eur J Cell Biol.

[58] Oppenheim HA, Lucero J, Guyot AC, Herbert LM, Mcdonald JD, Mabondzo A, Lund AK (2013). Exposure to vehicle emissions results in altered blood brain barrier permeability and expression of matrix metalloproteinases and tight junction proteins in mice. Part Fibre Toxicol.

[59] Suofu Y, Clark JF, Broderick JP, Kurosawa Y, Wagner KR, Lu A (2012). Matrix metalloproteinase-2 or -9 deletions protect against hemorrhagic transformation during early stage of cerebral ischemia and reperfusion. Neuroscience.

[60] Sarkar S, Mukherjee A, Das N, Swarnakar S (2017). Protective roles of nanomelatonin in cerebral ischemia-reperfusion of aged brain: matrixmetalloproteinases as regulators. Exp Gerontol.

