# Supplementary material for: IL-17A contributes to perioperative neurocognitive disorders through blood-brain barrier disruption in aged mice
Source: J Neuroinflammation. 2018 Nov 30;15:332. doi: 10.1186/s12974-018-1374-3 (PMC6267879; doi:10.1186/s12974-018-1374-3)
Supplement: Supplementary file 1 — Training and learning data. Table A. Freezing time before shock during the training period. Table B. Freezing time after shock 1 during the training period. Table C. Freezing time after shock 2 during the training period. Table D. Freezing time in contextual fear test. (n = 12). (PDF 73 kb) [file 12974_2018_1374_MOESM1_ESM.pdf]

Table A: Training data (before shock) (totally 100s)

	Con	Sur	Sur+anti-IL-17A	Sur+isotype
freezing time （s） (1)	1.59	0.17	2.62	1.54
freezing time （s） (2)	1.95	2.52	2.66	1.96
freezing time （s） (3)	0.29	1.62	1.94	1.51
freezing time （s） (4)	1.6	0.12	2.9	0.61
freezing time （s） (5)	1.14	0.28	1.47	1.22
freezing time （s） (6)	2.2	2.81	0.12	1.9
freezing time （s） (7)	0.67	1.87	0.21	0.45
freezing time （s） (8)	2.46	0.15	2.77	2.73
freezing time （s） (9)	0.5	0.61	1.95	1.3
freezing time （s） (10)	0.94	0.44	2.28	2.01
freezing time （s） (11)	2.1	0.21	2.5	0.16
freezing time （s） (12)	2.46	1.48	1.68	1.06
freezing time (%)(mean±sem）	1.492±0.22	1.023±0.29	1.925±0.27	1.371±0.21

Table B: Training data (after shock 1) (totally 100s)

	Con	Sur	Sur+anti-IL-17A	Sur+isotype
freezing time （s） (1)	11.78	7.92	9.88	8.09
freezing time （s） (2)	13.55	14.02	8.39	13.86
freezing time （s） (3)	7.48	8.35	10.01	9.49
freezing time （s） (4)	9.45	9.62	12.02	10.5
freezing time （s） (5)	14.46	9.46	12.24	10.86
freezing time （s） (6)	10.6	11.25	10.88	12.66
freezing time （s） (7)	7.21	11.13	8.29	8.73
freezing time （s） (8)	14.89	11.44	13.97	10.11
freezing time （s） (9)	14.22	13.88	13.54	10.65
freezing time （s） (10)	8.23	13.01	14.05	14.9
freezing time （s） (11)	9.59	14.6	13.54	8.38
freezing time （s） (12)	14.14	14.33	10.11	8.32
freezing time (%)(mean±sem）	11.3±0.84	11.58±0.69	11.41±0.61	10.55±0.64

Table C：Training data (after shock 2) (totally 100s)

	Con	Sur	Sur+anti-IL-17A	Sur+isotype
freezing time （s） (1)	24.91	24.81	17.09	25.57
freezing time （s） (2)	20.4	26.58	20.36	25.92
freezing time （s） (3)	26.94	18.98	26.54	22.21
freezing time （s） (4)	26.76	22.91	24.41	26.58
freezing time （s） (5)	18.23	26.78	25.33	19.87
freezing time （s） (6)	19.23	22.17	20.19	19.09
freezing time （s） (7)	21.53	19.5	24.44	18.4
freezing time （s） (8)	24.03	20.83	23.68	26.08
freezing time （s） (9)	22.52	19.33	24.97	24.57
freezing time （s） (10)	24.57	24.98	22.36	25.06
freezing time （s） (11)	18.54	26.08	20.93	26.3
freezing time （s） (12)	23.84	26.87	26.01	23.42
freezing time (%)(mean±sem）	22.63±0.88	23.32±0.90	23.03±0.83	23.59±0.86

Table D: Testing data (totally 300s)

	Con	Sur	Sur+anti-IL-17A	Sur+isotype
freezing time （s） (1)	65.25	44.37	51.54	49.41
freezing time （s） (2)	55.11	49.95	69.66	54.57
freezing time （s） (3)	64.08	38.88	62.01	34.92
freezing time （s） (4)	59.58	49.95	48.51	38.79
freezing time （s） (5)	62.82	52.74	62.82	35.73

freezing time（s） (6)	52.02	47.16	56.52	43.98
freezing time（s） (7)	60.03	46.71	50.64	45.69
freezing time（s） (8)	69.99	41.25	53.82	49.5
freezing time（s） (9)	58.71	35.94	64.05	44.28
freezing time（s） (10)	72.57	47.94	54.21	35.04
freezing time（s） (11)	66.09	57.12	53.88	30.84
freezing time（s） (12)	79.02	52.17	57.69	45.72
freezing time (%)(mean±sem）	21.26±0.73	15.67±0.59	19.04±0.61	14.12±0.70
