# Supplementary material for: IL-17A contributes to perioperative neurocognitive disorders through blood-brain barrier disruption in aged mice
Source: J Neuroinflammation. 2018 Nov 30;15:332. doi: 10.1186/s12974-018-1374-3 (PMC6267879; doi:10.1186/s12974-018-1374-3)
Supplement: Supplementary file 2 — Original full Western blotting images for Figs. 6 and 7. The cross-section was not included in this study. (PDF 4281 kb) [file 12974_2018_1374_MOESM2_ESM.pdf]

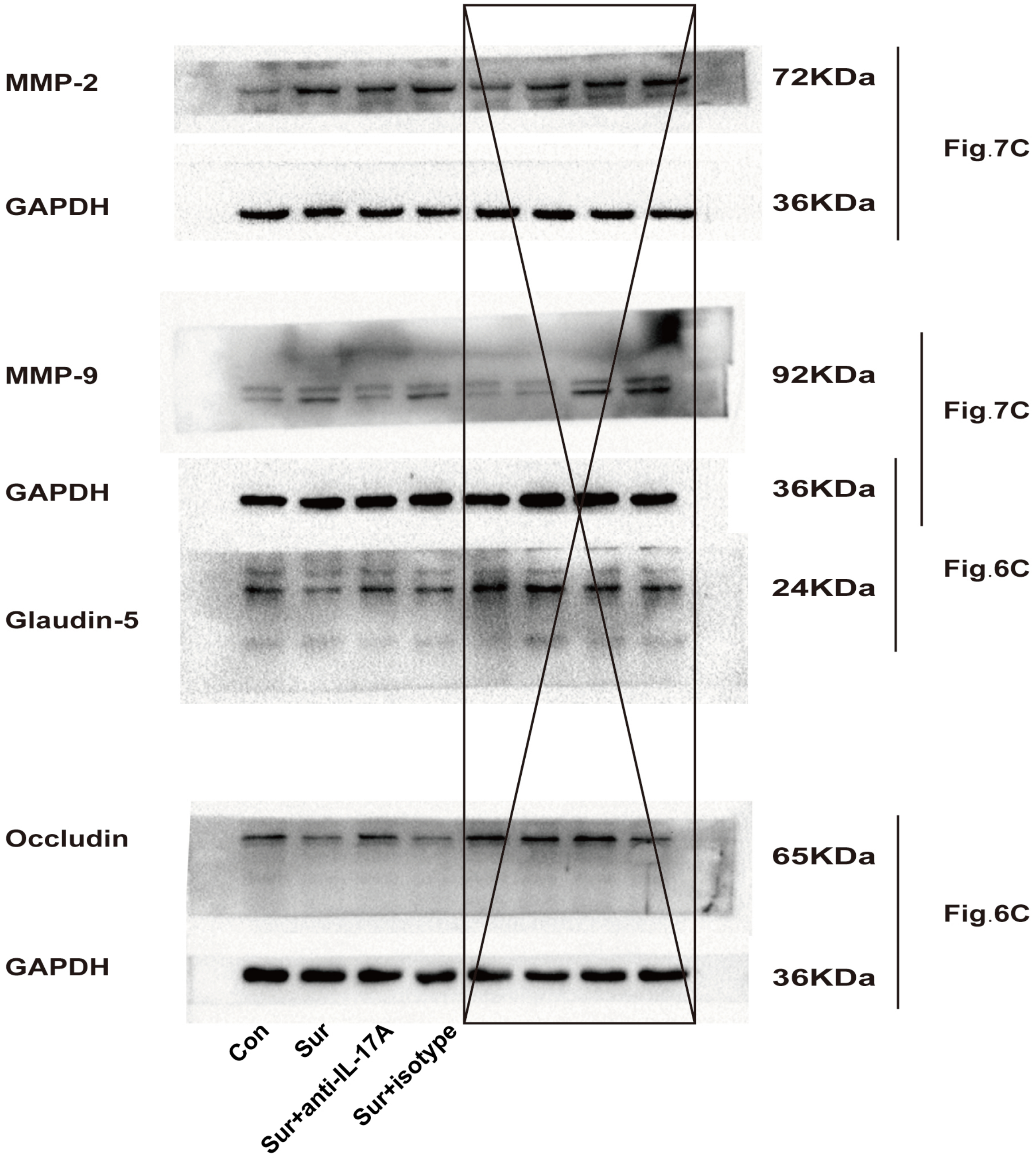

MMP-2
72KDa
GAPDH
36KDa
Fig.7C
MMP-9
92KDa
Fig.7C
GAPDH
36KDa
Glaudin-5
24KDa
Fig.6C
Occludin
65KDa
GAPDH
36KDa
Fig.6C
Con
Sur
Sur+anti-IL-17A
Sur+isotype
